# The Effect of Visual Stimuli on Stability and Complexity of Postural Control

**DOI:** 10.3389/fneur.2018.00048

**Published:** 2018-02-08

**Authors:** Haizhen Luo, Xiaoyun Wang, Mengying Fan, Lingyun Deng, Chuyao Jian, Miaoluan Wei, Jie Luo

**Affiliations:** ^1^Key Laboratory of Sensing Technology and Biomedical Instrument of Guangdong Province, Guangdong Provincial Engineering and Technology Center of Advanced and Portable Medical Devices, School of Engineering, Sun Yat-sen University, Guangzhou, China; ^2^Guangdong Work Injury Rehabilitation Center, Guangzhou, China

**Keywords:** virtual reality, balance control, entropy, center of pressure, head-mounted display

## Abstract

Visual input could benefit balance control or increase postural sway, and it is far from fully understanding the effect of visual stimuli on postural stability and its underlying mechanism. In this study, the effect of different visual inputs on stability and complexity of postural control was examined by analyzing the mean velocity (MV), SD, and fuzzy approximate entropy (fApEn) of the center of pressure (COP) signal during quiet upright standing. We designed five visual exposure conditions: eyes-closed, eyes-open (EO), and three virtual reality (VR) scenes (VR1–VR3). The VR scenes were a limited field view of an optokinetic drum rotating around yaw (VR1), pitch (VR2), and roll (VR3) axes, respectively. Sixteen healthy subjects were involved in the experiment, and their COP trajectories were assessed from the force plate data. MV, SD, and fApEn of the COP in anterior–posterior (AP), medial–lateral (ML) directions were calculated. Two-way analysis of variance with repeated measures was conducted to test the statistical significance. We found that all the three parameters obtained the lowest values in the EO condition, and highest in the VR3 condition. We also found that the active neuromuscular intervention, indicated by fApEn, in response to changing the visual exposure conditions were more adaptive in AP direction, and the stability, indicated by SD, in ML direction reflected the changes of visual scenes. MV was found to capture both instability and active neuromuscular control dynamics. It seemed that the three parameters provided compensatory information about the postural control in the immersive virtual environment.

## Introduction

Virtual reality (VR) has been used to improve balance in patients with stroke (1–3), and the usability and effectiveness have been examined (2–4). However, the mechanisms behind the new intervention and the effect of the virtual environment on balance control have not been fully understood.

The effect of vision information provided by immersive VR has been investigated with the experimental regime of comparing postural sway in the eyes-open (EO), eyes-closed (EC), and VR conditions (5–7). However, there has been no consistent conclusion yet. Horlings et al. showed that best stability was achieved in the EO condition, and a similar body sway was found in the EC and VR condition (5). However, Chiarovano et al. (6) and Robert et al. (7) reported no significant difference between the EO and EC conditions. In addition, it was found that the photo-rendered three-dimensional virtual environment did not increase body sway, but the optokinetic virtual scenes, such as a bundle of random dots moving in the same direction, will alter the postural control. In a word, how the visual stimuli generated by VR influence the postural control need further investigation.

Balance maintenance in the upright stance is a process of changing human body, by coordinating the muscle contractions, to make sure the center of mass (COM) of the body moving around the equilibrium position. The process of postural control has been widely investigated by means of a “motion capture system” (8) or a force plate (8, 9). With the latter apparatus, the center of pressure (COP) can be calculated with the reaction force data. To assess postural control, several parameters have been proposed and can be categorized into static parameters (e.g., position), dynamic parameters (e.g., velocity, root mean square, SD), and non-linear parameters [e.g., sample entropy, fuzzy approximate entropy (fApEn)]. SD of the COP displacement represents the average absolute displacement around the mean position of the COP trajectory (8), which has been employed by numerous researchers (10, 11). Velocity of the COP signal is said to describe the dynamic activity of the balance control by reflecting both the magnitude and frequency of the postural adjustment. Some investigations have shown its high reliability in anterior–posterior (AP) direction (8, 12). fApEn can be used to assess the irregularity of COP motion, and it might be a superior complexity measure in monotonicity and robustness to noise.

The aim of this study was to investigate the effect of visual information provided by VR on dynamic body sway in the upright stance. The virtual environments we used were optokinetic drums, rotating around different axes. 16 healthy subjects were enrolled in the investigation. When they put on the head-mounted display (HMD) device, they could see some black and white stripe-pairs moving in front of them, just like when they were sitting inside a large optokinetic drum. The reason we used the drum scene instead of the dot scene was that stripes were reported more effective in a virtual environment (13). The experiment included five kinds of visual input: EO, EC, and three different VR scenes. The parameters we used were mean velocity (MV), SD, and fApEn of the COP data.

## Materials and Methods

### Participants

Sixteen healthy subjects (11 females, mean age: 22 years, range: 20–24 years) were enrolled by advertisements. All the participants had normal or corrected-to-normal eyesight and reported no history of ocular or neuromuscular disorder or vestibular dysfunction that can alter their balance. They all signed the written informed consent about the purpose and procedures of the study prior to the experiments, and they were free to withdraw from the experiment at any time. Ethical approval in accordance with the Declaration of Helsinki was provided by Guangdong Work Injury Rehabilitation Center.

### Apparatus and Stimuli

**Figure 1:**
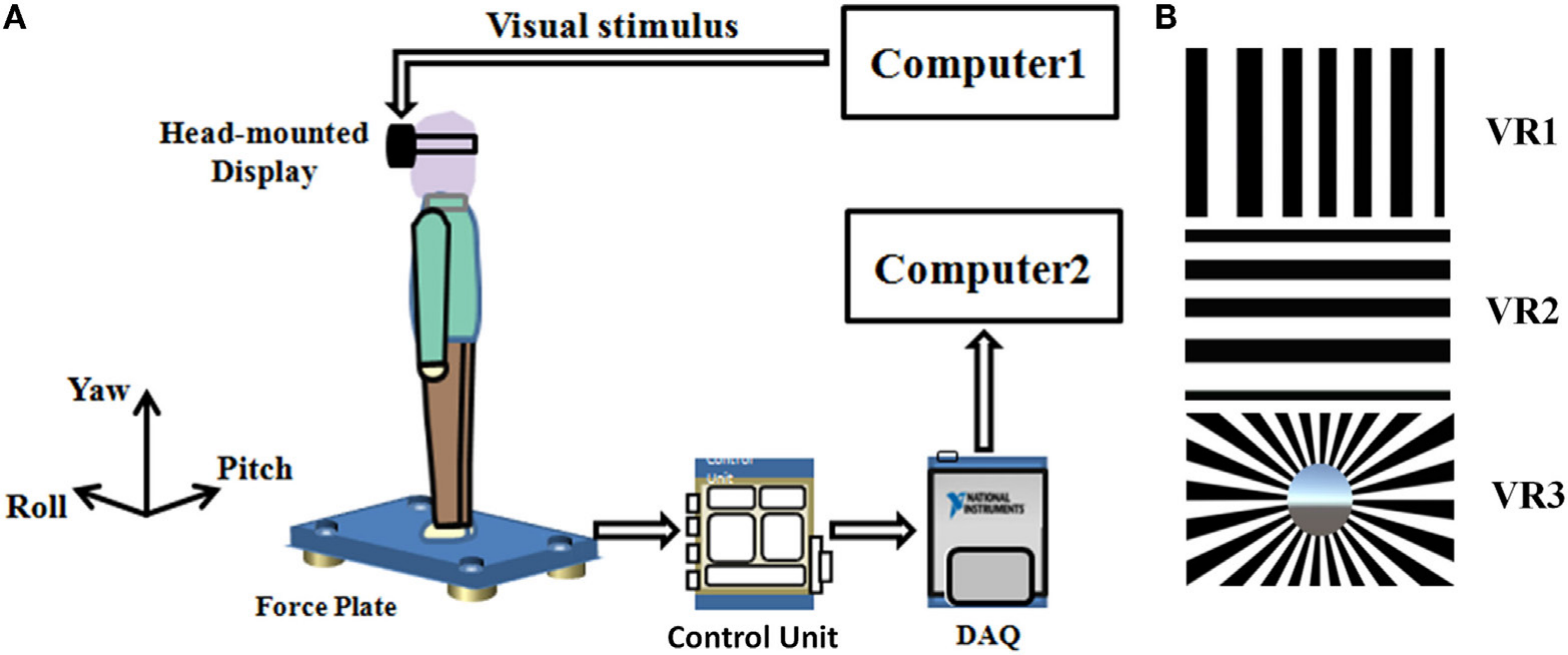
**(A)** Schematic diagram of experimental setup. Scenes generated using computer 1 and transmitted to head-mounted display (HMD). Subjects viewed scenes inside the (HMD) while standing on a force plate. The postural output is then amplified, collected by a multichannel data acquisition system (DAQ), and the data were sent to computer 2 for subsequent analysis. **(B)** Screenshots of the virtual optokinetic drum scenes around three coordinate axes from top to bottom: yaw (VR1), pitch (VR2), and roll (VR3).

The experimental devices included: (a) an HMD device (OculusRift Dk2, CA, USA) with a large field of vision (100°) and a high resolution LED screen (960 × 1,080 each eye), to show the VR scenes; (b) a desktop computer, with an Intel i7 CPU and an NVIDIA GTX 970 GPU, to create the VR scenes and drive the HMD device; (c) a multi-component force plate (Kistler type 9260, Kistler AG, Winterthur, Switzerland), placed stably on the ground to, to measure the change of force; (d) a control unit (Type 5233A2) with a built-in 8-channel charge amplifier equipped with filter bridges (cut-off frequency above to 7 kHz); (e) a data acquisition system, whose sampling frequency was set to 1,000 Hz; (f) a portable computer to record and analyze the data of the force plate (Figure 1A).

There were five types of visual exposure conditions: EC, eyes open (EO), and three VR conditions. In the VR conditions, an optokinetic drum was generated and rotated around a certain axis. When the participants put on the helmet, they saw some black and white stripe-pairs moving in front of them, just like when they were sitting inside a large drum, whose inner surface was painted with 24 equal-width black-and-white stripe pairs (14, 15). The drum could rotate around three different axes, i.e., yaw (VR1), pitch (VR2), and roll (VR3), with a constant speed (five rounds per minute) (Figure 1B). These VR scenes were created with the 3D Unity engine (version 5.3.3, Unity Technologies, CA, USA) and were displayed at a constant rate of 41 frames per second in the HMD.

### Experimental Procedures

To provide a comfortable experience to participants, we adjusted the HMD with individual’s pupil distance, measured with the built-in configuration utility, before the experiment. The experiment consisted of 10 trials, and one visual exposure condition for a trial. The five conditions were employed in a random order, but the same condition could not be employed in two adjacent trials, and each condition should be repeated twice. The duration of each visual exposure in a trial was 90 s, and there was a 1-min break between each two trials.

During the experiment, participants were required to stand on the force plate. Their feet should be put together, and their arms were required to be at their sides. In the EC condition, the subjects closed their eyes for the whole trial. In the EO trials, they were instructed to look at a fixed point in front of them. In the VR conditions, they put on the HMD device and were required to look straight ahead without attempting to follow the moving scenes. After the subject stood properly, the visual stimulus display and the data recording began synchronously.

### Data Analysis

**Figure 2:**
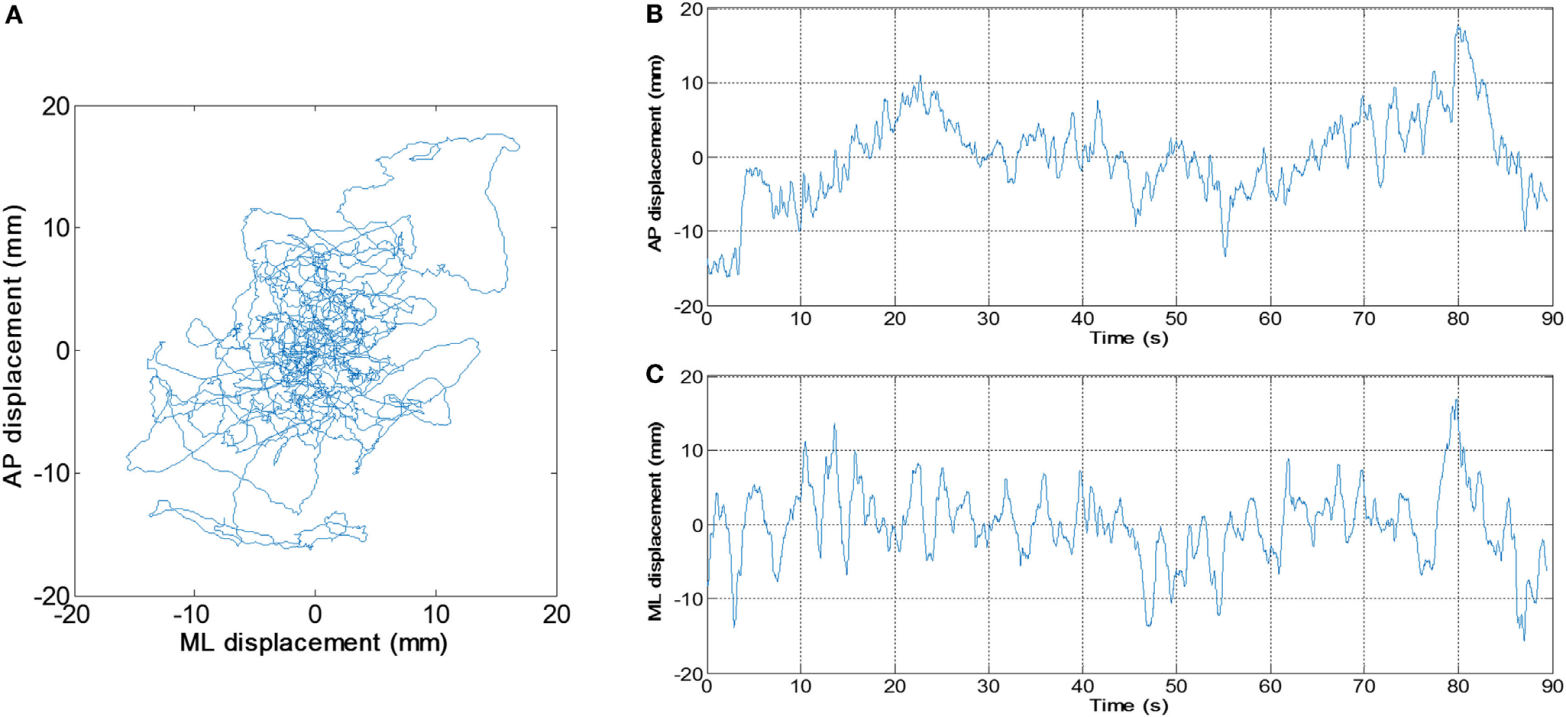
An example of center of pressure (COP) signal. **(A)** Planar COP trajectory. **(B)** Anterior–posterior and **(C)** medial-lateral displacement of COP varies with time, respectively.

An application was developed in our laboratory using MABLE 2016a (MathWorks, Natick, MA, USA) to perform the data analysis. The length of the raw data of each trial was 90,000. The raw data were digitally filtered with fourth order zero-lag Butterworth low-pass filter, whose cutoff frequency was set to 20 Hz (16). The (AP) displacement and medial-lateral (ML) displacement of the COP were derived from the filtered data (Figure 2). We calculated values of MV and SD as follows:

$${\text{SD}}_{\text{AP}}=\sqrt{\frac{1}{N}\sum_{i}^{N}{\left|{\text{AP}}_{i}-\mu_{\text{AP}}\right|}^{2}}$$

$${\text{SD}}_{\text{ML}}=\sqrt{\frac{1}{N}\sum_{i}^{N}{\left|{\text{ML}}_{i}-\mu_{\text{ML}}\right|}^{2}}$$

where AP_i_ and ML_i_ were the COP displacement time series in AP and ML directions, respectively; *N* was the data length, i.e., 90,000; μ_AP_ and μ_ML_ were the mean values of AP_i_ and ML_i_, respectively. SD_AP_ and SD_ML_ represented SD of COP signal in AP direction and ML direction, respectively.

We calculated MV of the COP signal in AP and ML directions as follows:

$${\text{MV}}_{\text{AP}}=\frac{1}{N-1}\sum_{i=1}^{N-1}\frac{\left|{\text{AP}}_{i+1}-{\text{AP}}_{i}\right|}{\text{T}}$$

$${\text{MV}}_{\text{ML}}=\frac{1}{N-1}\sum_{i=1}^{N-1}\frac{\left|{\text{ML}}_{i+1}-{\text{ML}}_{i}\right|}{\text{T}}$$

where T was the sampling interval.

We also calculated fApEn to assess the complexity of the COP signal (17, 18). Higher values of fApEn means lower repeatability of vectors and predictability in the COP time series. As suggested by Lestienne et al. (19), it is necessary for the COP time series to be down-sampled before calculating fApEn, because the effective bandwidth of the COP signal, containing physiological information is 0–10 Hz, and signal oversampling could cause artificial colinearities, hence affect the variability data (20). We down-sampled the filtered COP to 20 Hz. After down-sampling, the length of the filtered COP data was 1,800, and we calculated fApEn in the following steps.

Given an *N* point time series {*u*(i):1 < *i* < *N*}, a vector sequence $X_{i}^{m}$ could be derived:

$$X_{i}^{m}=\left\{u(i),\;\ldots \;,u(i+m-1)\right\}-\frac{1}{m}\sum_{j=0}^{m-1}\;u(i+j)$$

And the maximum absolute difference between two different vectors of $X_{i}^{m}$ and $X_{j}^{m}$, $d_{ij}^{m}$, could be calculated:

dijm=maxk∈(0,m−1)|u(i+k)−1m∑k=0m−1 u(i+k)−u(j+k)−1m∑k=0m−1 u(j+k)|(i,j=1,2, … ,N−m+1;  i≠j)

$D_{ij}^{m}$ (*n, r*) represented the similarity of the two vectors $X_{i}^{m}$ and $X_{j}^{m}$, and it could be obtained by the fuzzy membership function:

$$D_{ij}^{m}(n,r)=\exp\left(-{\left(\frac{d_{ij}^{m}}{r}\right)}^{n}\right)$$

Then, the following function averaged all of $D_{ij}^{m}$ yielding φ*^m^*:

$$\varphi^{m}(N,\;r)=\frac{1}{N-m+1}\sum_{i=1}^{N-m+1}\ln\left(\frac{1}{N-m+1}\sum_{j=1,j\neq i}^{N-m+1}D_{ij}^{m}\right)$$

Finally, fApEn (*m, n, r*) was calculated in the following way:

$$\text{fApEn}(m,n,r)=\varphi^{m}(n,r)-\varphi^{m+1}(n,r)$$

The parameter *m* was the vector length; *n* and *r* determined the similarity boundary (18). In this study, these parameters’ values were selected as follows: *m* = 2, *n* = 2, *r* = std × 0.2, where std was the SD of the down-sampled COP time series.

### Statistical Analysis

The data were tested for statistical significance using 2 × 5 repeated measures analysis of variance (ANOVA) with a Bonferroni *post hoc*. Within-subject factor was visual condition (EO, EC, VR1, VR2, and VR3), and between-subject factor was Direction (AP and ML). All statistical analysis were performed with SPSS (version 19.0.0), and *p* = 0.05 was used as the minimal significance level.

## Results

Significant visual condition effect was found in MV, SD, fApEn parameters (Table 1). No significant effect was found in direction or direction × visual condition interaction on all three parameters (Table 1).

**Table 1:** Results of two-way analysis of variance (MV, mean velocity; SD, standard deviation; fApEn, fuzzy approximate entropy).

Parameter		*F*	*p*
MV	Visual condition	**18.479**	**<0.001**
	Direction	1.178	0.286
	Direction × visual condition	1.699	0.193

SD	Visual condition	**10.332**	**<0.001**
	Direction	0.305	0.585
	Direction × visual condition	2.379	0.055

fApEn	Visual condition	**9.092**	**0.001**
	Direction	1.768	0.194
	Direction × visual condition	0.993	0.397

*Bold font represents significance at p < 0.05 level*.

**Figure 3:**
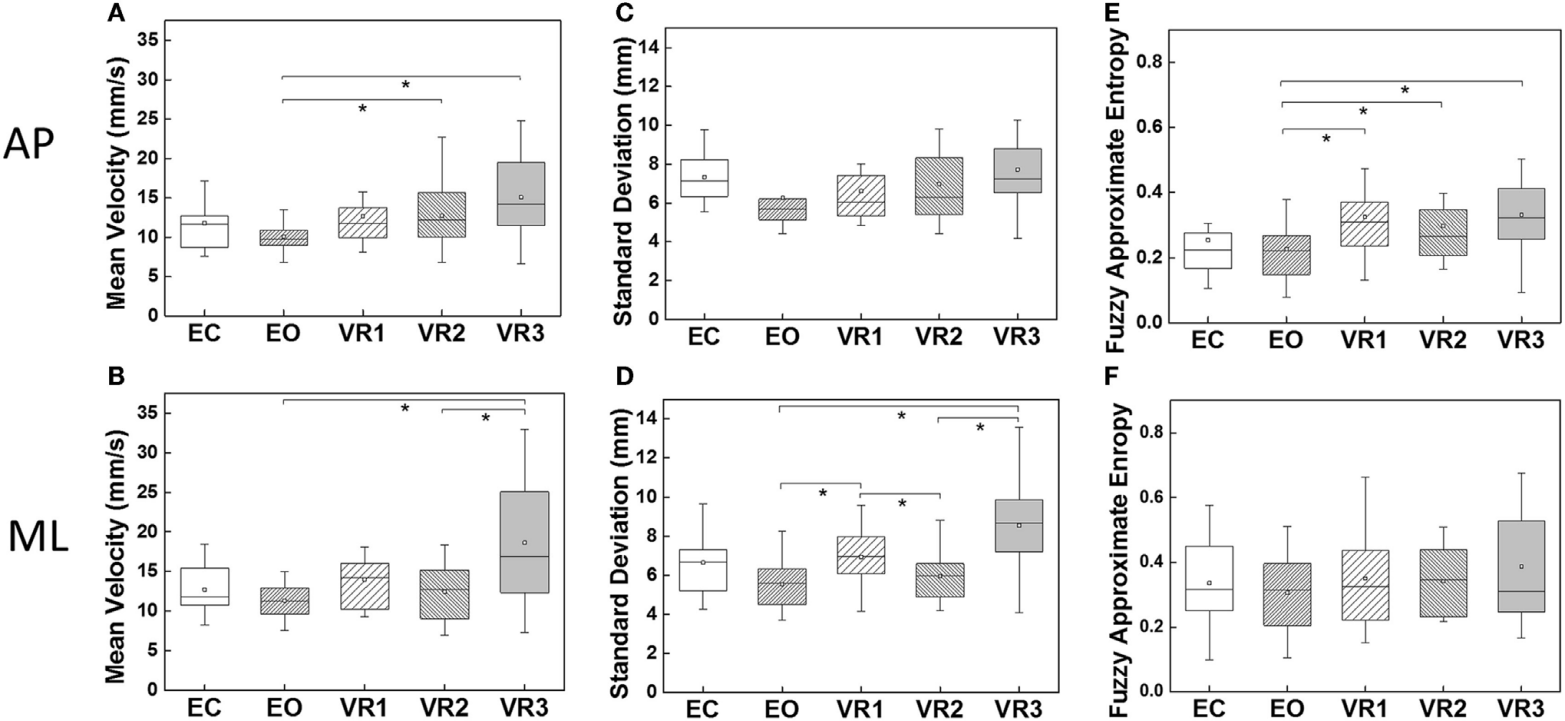
Comparisons of the five visual exposure conditions in terms of the three parameters in anterior–posterior (AP) and medial-lateral (ML) directions; **(A,B)** mean velocity (MV) in AP and ML direction, respectively; **(C,D)** SD in AP and ML direction, respectively; **(E,F)** fuzzy approximate entropy (fApEn) in AP and ML direction, respectively. The mean values of parameters are marked in squares. Asterisks denotes *p* < 0.05.

The box-whiskers-plots for MV, SD, as well as fApEn in both AP and ML directions are shown in Figure 3. A consistent trend could be observed in all three parameters, i.e., the lowest value was obtained in the EO condition, and the largest was in the VR3 condition. In addition, none of the parameters showed statistical difference when comparing the EC and EO conditions.

In AP direction, values of MV and fApEn in the VR2 and VR3 conditions were significantly higher than that in the EO condition (EO-VR2: *p* = 0.04 for MV, *p* = 0.004 for fApEn; EO-VR3: *p* = 0.003 for MV, *p* = 0.004 for fApEn). fApEn was noticeably larger in the VR1 condition than in the EO condition (*p* = 0.008). There was no statistical significance between each two conditions for SD.

In ML direction, MV in the VR3 condition was significantly larger than that in the EO (*p* = 0.01) and VR2 (*p* = 0.04) conditions. SD in the VR1 condition was remarkably greater than in the EO (*p* = 0.03) and VR2 (*p* = 0.018) conditions. And SD in the VR3 condition was significantly larger than that in the EO (*p* = 0.001) and VR2 (*p* = 0.002) conditions. Therefore, SD discriminated different Visual Conditions better in ML direction than in AP direction. However, fApEn yielded similar results for all visual conditions in ML direction.

## Discussion

The main objective of this work was to investigate how the visual feedback and different dynamic visual perturbations in a HMD affected the postural control of healthy young participants during upright stance in both AP and ML directions. In our study, MV and SD of the COP, measured with a force plate, were utilized to assess the dynamic behavior of the balance control, and fApEn was used to measure the complexity of the COP signal.

### Trends in the Parameters

Although not every comparison of two visual exposure conditions showed significant difference, the three parameters presented a similar trend: the lowest value was obtained in the EO condition, and the largest value was obtained in the VR3 condition, no matter in which COP direction. Previous studies (6, 19, 21) have found MV and SD of COP were lower in EO condition, compared with EC condition, demonstrating the contribution of visual information to balance maintenance. Our results were in accordance with previous reports. The effect of VR scenes on postural stability could be investigated with inertial sensors, such as gyroscopes (5), and it was found that the postural sway, in terms of shoulder sway angle and its velocity, when viewing simulated 3D-VR scenes was similar to that when standing still with EC but significantly larger than that when standing still with EO. However, two recent studies (6, 7) reported that, in terms of COP parameters, data obtained were similar in the EO and EC conditions, and static filmed 3-dimensional virtual environment would not add unstable factor, compared to the EO condition, while simulated optokinetic scenes indeed affected the postural control. In our experiment, besides of amplitude of the COP signal, we also employed velocity and non-linear parameter, i.e., fApEn, to characterize the postural control performance. The three parameters yielded similar results in the EO and EC conditions, and our optokinetic drum scenes affected MV in both directions, SD in ML direction, and fApEn in AP direction. Our results were in agreement with those in the optokinetic dots experiment (6). The EO and EC conditions yielded similar fApEn, which was consistent with previous study, reporting that closing eyes did not produce striking effect on the complexity of postural control system among young people (22).

### Meanings of the Parameters

Traditionally, MV and SD of the COP signal were considered as dynamic characteristics of balance control, and fApEn measured the regularity and complexity of the system. In detail, SD was said to characterize the spread of the COP amplitude, indicating postural instability, MV was considered as a more reliable quantity containing both spatial and frequency information of the COP signal (23) and fApEn revealed the coordinated muscle contractions that constrained the COM around the equilibrium position (24). In general, larger COP displacements (larger SD) would be companied with faster COP adjustments (higher MV) for the sake of balance maintenance, and this relationship could also be observed in our experiment. On the other hand, slower COP adjustment (lower MV) was considered to be associated with sensory feedback control (7), and sensory-input was assumed to increase neuromuscular intervention, hence generating a larger fApEn (24). However, this relationship was not observed in our experiment. Instead of considering MV as a parameter reflecting the cause of balance maintenance, such as sensory feedback, we will discuss an alternative interpretation that it was a measure of the performance of body adjustment due to balance control.

We started from the interesting opposing discrimination ability of SD and fApEn: SD was more discriminant in ML direction, and fApEn was more discriminant in AP direction. Since “the COP is the neuromuscular response to the imbalances of the body’s COM” (25), the fApEn should reflect the complexity of the neuromuscular response. Our results implied that the balance control in ML direction was more or less in the same pattern since the fApEn means for the five visual exposure conditions were similar, while the system complexity measures in AP direction fell into two levels, one for the EO condition, and the other for the VR condition. It suggested that the active neuromuscular intervention was more diverse and adaptive in AP direction. Opposed to fApEn, SD of COP amplitude was similar in AP direction but different in ML direction in our experiment. It seemed that the similar stable state in AP direction was associated with the multi-level active neuromuscular regulation complexity, while the spread of COP amplitude in ML direction could not be well controlled with similar neuromuscular regulation strategies under different visual exposure. In summary, optokinetic virtual scenes induced a more complex neuromuscular response in AP direction, and affected the stability in ML direction. Since the discrimination ability of MV was similar to fApEn, in AP direction, and SD, in ML direction, we speculated that MV was a measure of movement performance, reflecting both the active neuromuscular response and the instability induced by visual perturbation.

### Influences of Visual Scenes

Abundant studies have revealed that visual information contributes to balance maintenance, but visual information could also be a perturbation to balance control. Here, we categorized visual scenes into physical and virtual ones, and static and dynamic ones. The widely used EO condition relates to the static physical scenes, since in most experiment, including ours, the participants were instructed to fix their sights to somewhere in front of them. An example of dynamic physical scene is realized by a “Swinging Room” apparatus (26), which is a large box suspended on four ropes above the floor, and it could swing along the ropes to provide a physical moving scene for a person standing still inside the box. In the experiment of ref (7), a static virtual scene was generated by rendering a photo into a three-dimensional one. The dynamic virtual scenes in the HMD always provide an immersive VR experience. The simplest kind of dynamic virtual scenes is optokinetic simulation (6, 13). In our experiment, we generated an optokinetic drum, which could rotate around different axes.

When standing on a firm platform, the static visual information could not affect the postural control, but the dynamic scenes induced postural sway, no matter whether it is physical or virtual (6, 7, 26). Our results also supported this claim. In addition, our results implied a direction-related influence of the dynamic virtual scenes. In our experiment, in the VR1 condition, the vertical stripes on the optokinetic drum were moving horizontally; in the VR2 condition, the horizontal stripes were moving vertically; in the VR3 condition, the radial stripes were moving in a counterclockwise direction. MV of the VR2 condition was significantly higher than that of the EO condition in AP direction, but not in ML direction, suggesting an induced body sway in AP direction by the VR2 scene. SD of the VR1 condition was significantly larger than that of the EO and VR2 condition in ML direction but not in AP direction, suggesting a modulation of body sway was induced by the VR1 scene. Previous literature also reported that the postural displacement induced by the motion of the visual scene was in the same direction as stimulus (27–29). The velocity of the visual scenes in the literatures ranges from 0.02 and 0.16 (18) to 1/3 Hz (30). Our study showed that this modulation could also be found in a faster moving visual scene (5 rounds/s × 24 pairs/round = 2 pairs/s, i.e., 2 Hz). Since the VR3 scenes contain more complex optic flow information that moving both in horizontal and vertical direction, MV of the VR3 condition was larger than the EO condition in both AP and ML direction. The direction-modulated postural sway in our experiment supported that vision is a source of proprioceptive information for balance control (26), no matter whether it is in physical or virtual environment.

## Conclusion

We have shown that dynamic virtual environment could induce active neuromuscular regulation and instability. The parameters we have used to characterize balance control were MV, SD, and fApEn of the COP signal, measured with a force plate. We have demonstrated that fApEn revealed active neuromuscular regulation taking place mainly in AP direction, and MV was a measure indicating both active neuromuscular intervention and instability. These COP parameters should benefit quantification of the balance recovery.

### Additional Requirements

For additional requirements for specific article types and further information, please refer to Author Guidelines.

## Ethics Statement

All the participants signed the written informed consent about the purpose and procedures of the study prior to the experiments, and they were free to withdraw from the experiment at any time. Ethical approval in accordance with the Declaration of Helsinki was provided by Guangdong Work Injury Rehabilitation Center.

## Author Contributions

HL conducted most of the experiments, collected and analyzed the data, interpreted the results, and finished the draft manuscript. MW designed the virtual scenes and part of the experiment, and finished part of the draft. JL designed the study, helped to analyze the data, and interpret the results and revised the manuscript. JL and MF participated in the data collection and analysis. LD conducted part of the experiments, recruited the subjects, collected data. XW designed the study, conducted the experiments, and revised the manuscript. CJ participated in the data collection.

## Conflict of Interest Statement

The authors declare that the research was conducted in the absence of any commercial or financial relationships that could be construed as a potential conflict of interest.
